# State-of-the-art imaging for glioma surgery

**DOI:** 10.1007/s10143-020-01337-9

**Published:** 2020-06-30

**Authors:** Niels Verburg, Philip C. de Witt Hamer

**Affiliations:** 1 Department of Neurosurgery and Cancer Center Amsterdam, Amsterdam UMC location VU University Medical Center, Amsterdam, The Netherlands; 2 Division of Neurosurgery, Department of Clinical Neurosciences, Cambridge Brain Tumor Imaging Laboratory, University of Cambridge, Addenbrooke’s Hospital, Hill Rd, Cambridge, CB2 0QQ UK

**Keywords:** Glioma, Extent of resection, Imaging, Brain functionality, Neurosurgery

## Abstract

Diffuse gliomas are infiltrative primary brain tumors with a poor prognosis despite multimodal treatment. Maximum safe resection is recommended whenever feasible. The extent of resection (EOR) is positively correlated with survival. Identification of glioma tissue during surgery is difficult due to its diffuse nature. Therefore, glioma resection is imaging-guided, making the choice for imaging technique an important aspect of glioma surgery. The current standard for resection guidance in non-enhancing gliomas is T2 weighted or T2w-fluid attenuation inversion recovery magnetic resonance imaging (MRI), and in enhancing gliomas T1-weighted MRI with a gadolinium-based contrast agent. Other MRI sequences, like magnetic resonance spectroscopy, imaging modalities, such as positron emission tomography, as well as intraoperative imaging techniques, including the use of fluorescence, are also available for the guidance of glioma resection. The neurosurgeon’s goal is to find the balance between maximizing the EOR and preserving brain functions since surgery-induced neurological deficits result in lower quality of life and shortened survival. This requires localization of important brain functions and white matter tracts to aid the pre-operative planning and surgical decision-making. Visualization of brain functions and white matter tracts is possible with functional MRI, diffusion tensor imaging, magnetoencephalography, and navigated transcranial magnetic stimulation. In this review, we discuss the current available imaging techniques for the guidance of glioma resection and the localization of brain functions and white matter tracts.

## Introduction

**Fig. 1:**
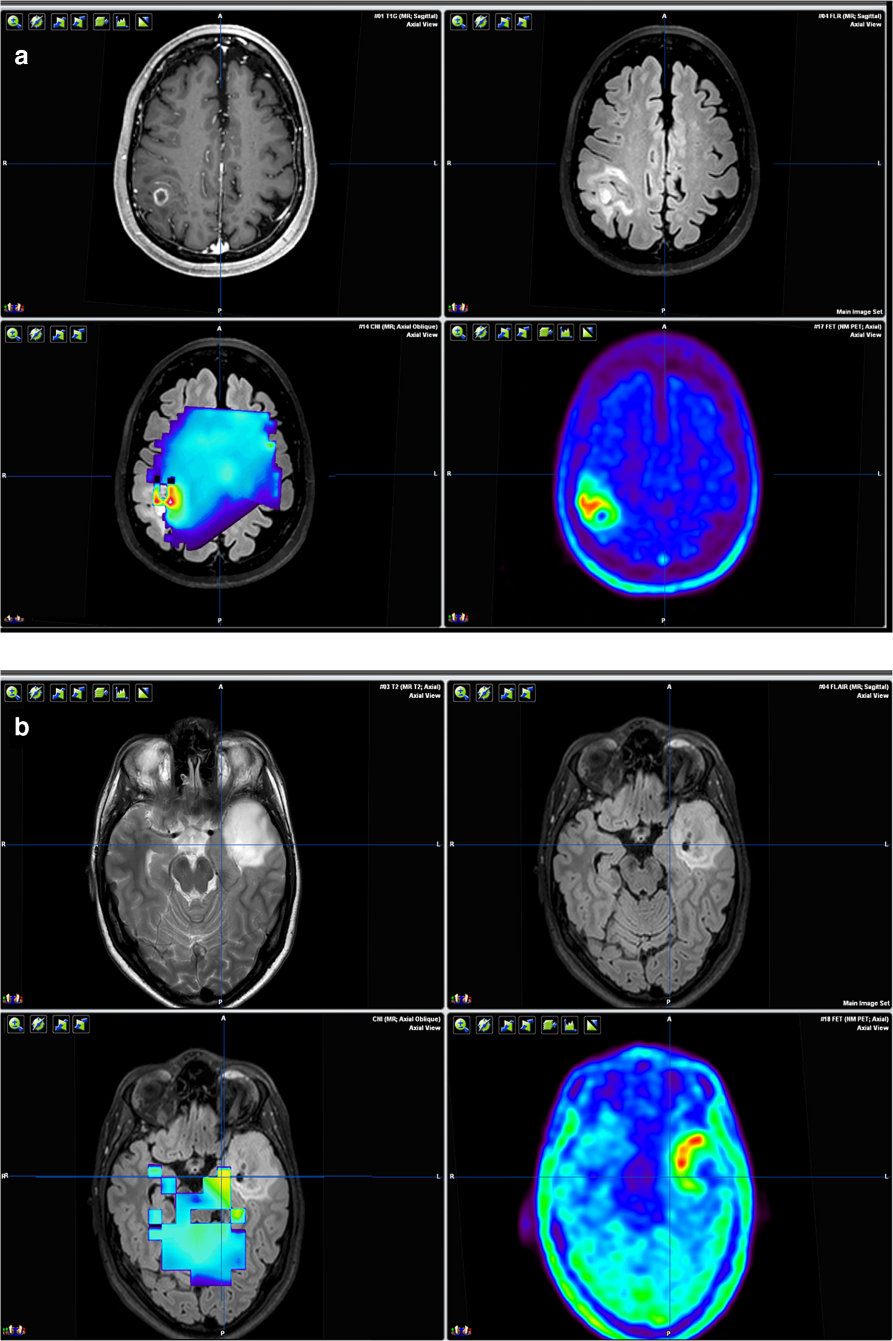
Exemplary standard and advanced imaging. **A** Patient with a right parietal enhancing glioblastoma, IDH-wild type. Upper left: T1-weighted MRI with a gadolinium-based contrast agent, Upper right: FLAIR MRI, lower left: multivoxel MRSI CNI projected on the FLAIR MRI. Lower right: amino acid ([^18^F]FET) PET. **B** Patient with a left temporal non-enhancing diffuse astrocytoma, IDH-mutant. The crosshair is projected to indicate the region with MRSI CNI and PET abnormalities just outside the FLAIR MRI abnormalities. Upper left: T2-weighted MRI, Upper right: FLAIR MRI, Lower left: multivoxel MRSI CNI projected on the FLAIR MRI, Lower right: amino acid ([^18^F]FET) PET

Surgical resection is the first treatment in the majority of patients with a diffuse glioma. Surgery aims at providing adequate tissue for diagnosis, relieving mass effect and achieving cytoreduction. To achieve maximal cytoreduction, pursued to improve patient’s survival [8, 86], the neurosurgeon needs to identify glioma infiltration during surgery. This is difficult due to the diffuse dissemination of glioma cells in the normal brain. The most widely used aid for the detection of glioma infiltration during surgery is imaging. Standard magnetic resonance imaging (MRI), T1-weighted gadolinium-enhanced (T1G) for enhancing gliomas (Fig. 1A), and T2 (T2w) or fluid attenuation inversion recovery (FLAIR) weighted for non-enhancing gliomas (Fig. 1B) are recommended [18].

These standard MRI sequences, however, are less accurate for the detection of glioma infiltration than advanced MRI sequences and imaging modalities [93]. Therefore, advanced imaging, as well as the use of intraoperative fluorescence, holds the potential to expand the resection beyond the standard MRI abnormalities, which may improve patient’s outcome [37, 52, 68, 71, 72, 89].

When expanding the resection, one must be aware of the potential loss of brain function due to the infiltration of tumor cells in normal functioning brain. Important brain functions, such as motor function, language, and neurocognitive functioning, need to be preserved since severe morbidity is not only associated with a decline in quality of life but also with survival [19]. The current standard to identify brain functions and white matter tracts is intraoperative direct cortical stimulation (DCS), a technique that provides an electrical stimulation to accomplish local excitation or inhibition in the cortex or white matter tracts that will result in a functional response [15]. Multiple techniques are available for the localization of brain functions or white matter tracts.

The neurosurgeon’s goal is to find the balance between the optimal oncological outcome by maximizing the resection and preventing severe morbidity by loss of brain functionality. Here, we discuss the state-of-the-art imaging techniques to guide glioma resection and imaging techniques to localize functions and white matter tracts, in order to achieve a maximal safe resection.

## Imaging techniques for the guidance of glioma resection

### Pre-operative imaging

#### Standard MRI

The current standard MRI sequences for the guidance of glioma resection have historically grown into use since their widespread availability. Clinical trials supporting the use of standard MRI for the guidance of glioma resection are lacking. Therefore, we discuss the indirect evidence for these sequences. This evidence comes from studies that investigated the effect of the extent of image-guided glioma resection on survival.

In enhancing glioma, the strongest evidence for the use of T1G MRI comes from a post hoc analysis of 243 patients from a randomized controlled trial (RCT), comparing fluorescence-guided surgery with standard neuronavigation [69, 89]. In this study—after correction for tumor size, edema, midline shift, location, age, Karnofsky Performance Scale and National Institutes of Health Stroke Scale—complete resection of contrast enhancement on post-operative T1G MRI, compared to incomplete resection, resulted in longer OS (16.7 versus 11.8 months, *p* < 0.01) [69].

In non-enhancing glioma, the choice for T2w or FLAIR MRI aided surgery depends on the surgeon’s preference, since direct comparison is lacking, which is reflected in the used imaging sequences in a recent review [2], where T2w, FLAIR, and T2w or FLAIR MRI were respectively used in 36%, 46%, and 18% of the studies. A possible benefit of FLAIR MRI is the suppression of the water signal intensity, which allows for better contrast of tumor in periventricular areas. Both T2w and FLAIR MRI aided resections are supported by retrospective studies [33, 86]. These studies prove the goal of complete resection of the standard MRI abnormalities in both enhancing and non-enhancing gliomas. The lack of studies directly comparing standard MRI- versus other imaging-guided resection, however, makes it impossible to judge if standard MRI is the best option for the guidance of glioma resection. Considering the evidence of diagnostic accuracy studies [93], more is to be expected from other MRI sequences or imaging modalities.

#### FLAIR MRI in enhancing glioma

In the majority of enhancing glioma, FLAIR abnormalities expand beyond the regions with contrast enhancement (Fig. 1A) [30]. These surrounding FLAIR abnormalities are sometimes addressed as peritumoral edema; however, many studies have proven the presence of glioma cells within these regions [24, 27, 38, 39, 76]. Extending the resection beyond contrast-enhanced regions using FLAIR has shown great potential. A large (*n* = 643) retrospective study found an improved OS for a more extensive (≥ 53%) resection of the surrounding FLAIR abnormalities after complete resection of contrast-enhanced regions, compared to less extensive resections (median OS 20.7 and 15.5 months, respectively; *p* < 0.01). Remarkably, a more extensive resection resulted in a lower complication rate (18% versus 26%, *p* = 0.04), which reflects, according to the authors, the increased use of DCS and imaging to visualize brain functions and white matter tracts. These promising results are a bit tempered by the fact that an extensive resection was only achieved in 25% of the patients [52]. Both FLAIR resection threshold, number of patients receiving extensive resection, and the lower complication rate with more extensive resection were confirmed in another study with 282 patients [68]. These are the largest studies comparing, although not randomized and prospective, different MRI sequences to aid glioma resection, therefore providing the strongest evidence for the use of other sequences than the current standard.

#### Magnetic resonance spectroscopy imaging

Magnetic resonance spectroscopy imaging (MRSI) measures biochemical components of a region of interest, which can be used to calculate, among others, the choline-*N*-acetyl aspartate index (CNI) to detect glioma (Fig. 1 A and B). The only study describing MRS-aided surgery reported an extended resection beyond contrast enhancement in 86% of seven enhancing gliomas and beyond FLAIR MRI abnormalities in 88% of eight non-enhancing gliomas. The target volume for resection was based on the lowest CNI threshold that allowed a safe resection, defined by functional imaging and anatomy. The survival benefit in this study is not clear due to the limited follow-up of 1 year, in which one enhancing glioma and none of the non-enhancing gliomas recurred [101]. A limitation of MRSI is the technical difficulty of obtaining a good-quality 3D MRS image due to the artifacts of non-brain tissue [51]. The concept of different threshold-based target volumes, as well as the possibility to aid resections beyond FLAIR abnormalities, makes MRSI an interesting technique that deserves further research.

#### Positron emission tomography

Positron emission tomography (PET) is a nuclear imaging technique that uses radioactive tracers to visualize perfusion, proliferation, metabolism, and neurotransmitters (Fig. 1 A and B). Multiple tracers are available for glioma imaging of which only the amino acid L-[methyl-11C]methionine (MET) is used to aid glioma resection. The only group reporting MET PET aided resection selected gliomas with ill-defined borders or enhancing gliomas with T2w or FLAIR abnormalities beyond the contrast enhancement. Two strategies were used for these gliomas: (1) to extend the resection beyond standard MRI abnormalities or (2) a focused resection of the most metabolic active parts of the tumor if a complete resection of MRI abnormalities was not possible. In enhancing gliomas, each strategy was achieved in one-third of patients, while PET was not contributive in the remaining one-third of patients. In non-enhancing gliomas, the first strategy was achieved in 74%; the second strategy in 14% and 12% of patients did not have a contributive MET PET. OS in enhancing gliomas was predicted by complete resection of MET uptake, achieved in 56% of patients, while complete resection of contrast enhancement, achieved in 35% of patients, did not [71]. Unfortunately, survival data was not collected. Limitations of PET imaging are the costs, an estimated 1600–2100 dollars for one scan [40], although a cost-effectiveness analysis showed that the use of MRI and PET is cost-effective [29]. Other limitations are the necessity of an on-site cyclotron for tracers with a short half time, and one-third of the non-enhancing gliomas are amino acid PET negative [49]. Although these retrospective results are biased by the specific inclusion criteria and the low percentage of complete resection of contrast enhancement, they show the potential of PET-aided glioma surgery.

#### Limitations pre-operative imaging

Three limitations of all pre-operative imaging are interobserver variation for tumor delineation, image fusion and registration setup inaccuracy, and inability to compensate for intraoperative surgery-induced changes. Interobserver variation for the delineation of gliomas is the difference in tumor volumes, as assessed on imaging, between different interpreters. In enhancing gliomas, this in only a minor issue since observer agreements are good (range 0.97–0.99) [47, 95]. In non-enhancing gliomas, however, agreements are considerably lower (range 0.48–0.77) for both T2w and FLAIR MRI [4, 95]. Possible causes for this lower agreement are the interpretation of the hyperintense T2w and FLAIR signal as edema or glioma infiltration and the less well-defined borders of the T2w and FLAIR abnormalities [95]. MRSI and PET are less limited by interobserver variability due to their quantitative analysis and the use of a threshold. Image fusion and registration setup inaccuracy occurs due to the translation of pre-operative images to the intraoperative situation. Since the intraoperative navigation is based on the 3D model of one MRI sequence, mostly 3D T1G or 3D FLAIR MRI, all other images that are used for the delineation need to be fused with this 3D sequence. This fusion is mostly performed with a linear method and inaccuracies of 1.0 to 3.0 mm have been reported [31]. Registration inaccuracy occurs due to the translation of the 3D MRI model to the actual patient. Depending on setup, inaccuracy varies between 1.59 and 3.86 mm [88]. The last but foremost limitation is the inability of pre-operative imaging to adjust to the new situation after surgical induces changes such as brain shift, tissue deformation, and tissue removal. Shifts between 7 mm inward and 8 mm outward were found after dura opening, and 9.7 mm inward and 15 mm outward (mean 2.7–5.4 mm) after tumor resection [62, 80]. The influence of these effects on the resection can be limited by circumscribing the tumor, instead of piecemeal removal, thereby limiting the brain shift.

### Intraoperative imaging

#### Intraoperative MRI

**Fig. 2:**
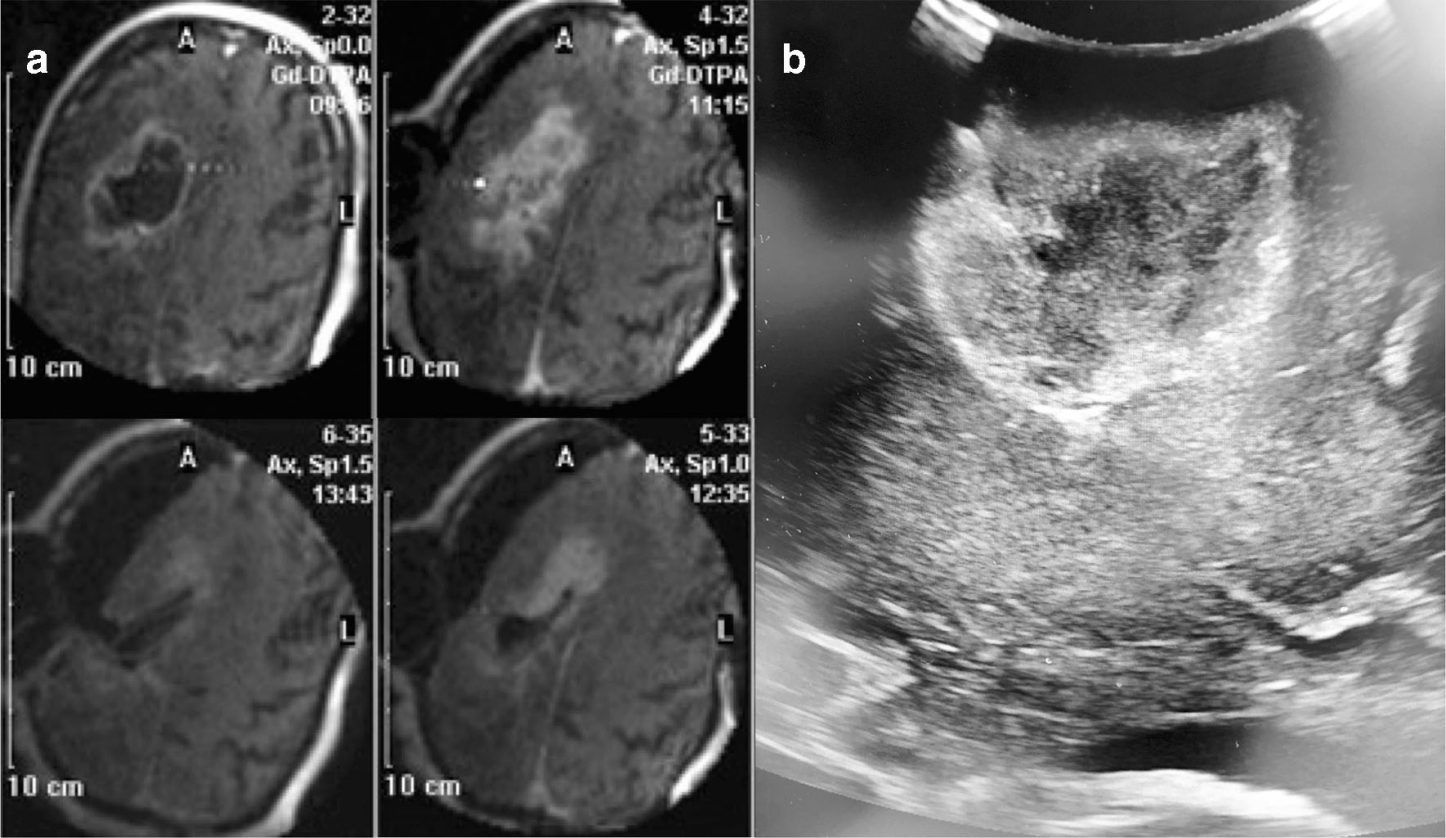
Examples of intraoperative imaging. **A** Intraoperative MRI of an enhancing right frontal glioblastoma with clockwise images of the progression of the resection with clear brain shift. Image courtesy of Dr. P Kubben [46]. **B** Intraoperative ultrasound of a left parietal glioblastoma

Intraoperative MRI (iMRI) has the advantage over pre-operative MRI that it can overcome its above-mentioned limitations. Since the images are acquired in the same position as the surgery, registration inaccuracy is lower for iMRI than pre-operative MRI [91]. Even more important, iMRI can visualize the altered anatomy due to intraoperative changes, which reduces their influence on navigation inaccuracy (Fig. 2A) [57].

Besides these advantages in navigation accuracy, iMRI allows for the detection of residual tumor after a first attempt for a maximal resection. In enhancing glioma, a RCT with 49 patients found a higher percentage of complete resections of contrast enhancement in the iMRI group (96% versus 68%, *p* < 0.01) [85], although the effect on survival is still awaited for. In non-enhancing glioma, multiple retrospective studies report improved complete resection rates (14 to 19%) of T2w or FLAIR abnormalities using iMRI [58, 63, 67]. Two major drawbacks of iMRI are the high initial costs, 3.8 million dollars for the ultra low-field model in 2011, and the prolonged duration of the surgery, up to 2 h, due to scan time [48, 55]. Although iMRI has proven its value for the purpose of complete resection of standard MRI abnormalities, studies using iMRI to extend the resection beyond these standard imaging abnormalities are lacking. Even if this is possible, alternatives could be considered due to the high cost and prolonged surgical time of iMRI.

#### Ultrasound

The visualization of returning sound waves can be used to detect glioma by direct application of the ultrasound (US) probe on the tissue (Fig. 2B). Like with iMRI, this results in real-time imaging that is not influenced by navigation inaccuracy or intraoperative changes. US-guided resection achieved a complete resection of US abnormalities in 67% of the 61 enhancing and 61% of the 51 non-enhancing glioma patients. This resulted in an improved 2-year OS compared to a random selected control group (32.8% versus 13.3% and 88.2% versus 53.3%, both *p* < 0.05, respectively enhancing and non-enhancing glioma) [97]. New US techniques like high-frequency linear probes and ultrasonic contrast are introduced and hold potential to maximize resections compared to standard US [10, 75]. Direct comparison of high frequency US with iMRI, using biopsies from residual tumor and normal control sites after resection, resulted in a significantly higher sensitivity for US (sensitivity 76% versus 55%, *p* = 0.021) and not significant difference in specificity (specificity 74% versus 58%) [11]. Besides detection of glioma tissue, US can be used to update the pre-operative MRI-based neuronavigation [74]. Limitations of US are the training necessary to create a good-quality image; problems with artifacts due to blood, hemostatic material, bone and other structure material; and the 2D aspect of US. This makes the acquisition and interpretation of US for glioma delineation challenging [83]. Still, US can serve as a good and cheaper alternative for iMRI.

#### Fluorescence

**Fig. 3:**
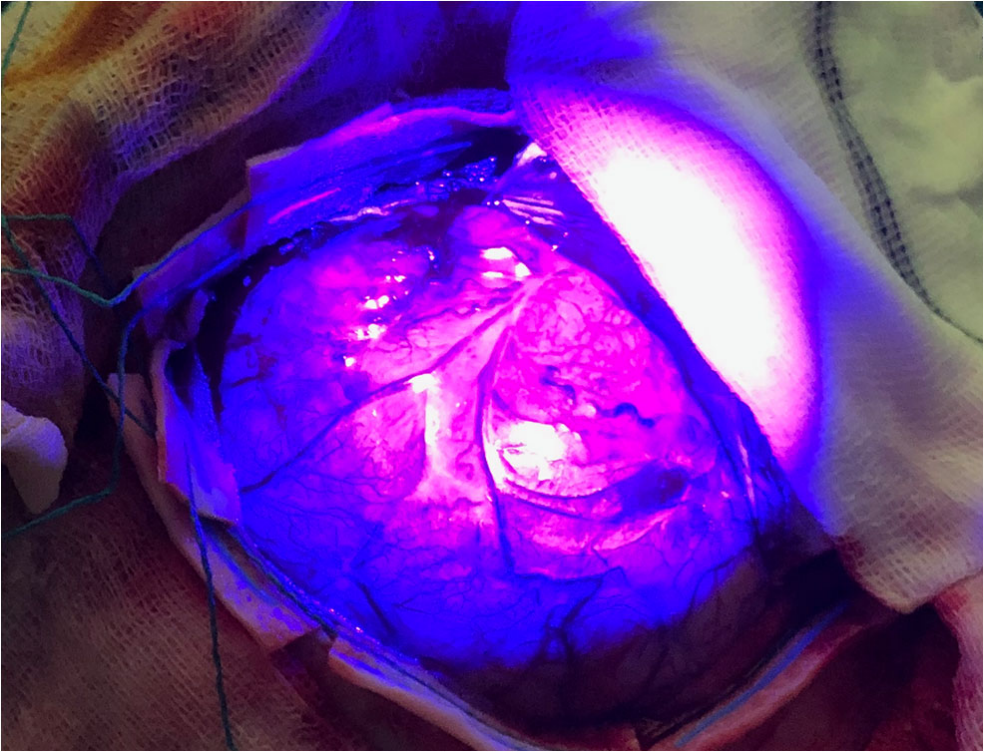
Example of intraoperative fluorescence. Intraoperative image of 5-ALA-guided resection of superficial glioblastoma with clear pink fluorescence of the tumor with the surrounding normal tissue appearing blue

Although not a standard imaging technique per se, the use of fluorescence during glioma resection aids the surgeon in the visualization of the tumor. Multiple agents are available for intraoperative fluorescence of which 5-aminolevulinic acid (5-ALA) and sodium fluorescein (SF) are most common in daily practice. 5-ALA is a non-fluorescent prodrug that leads to intracellular accumulation of fluorescent porphyrins in malignant gliomas. These porphyrins can be visualized intraoperatively with a special filter for the operating microscope resulting in a pink appearance of the tumor, while the normal tissue appears blue (Fig. 3). A large RCT (*n* = 322) reported higher GTR rates of contrast enhancement (65% versus 36%, *p* < 0.0001) and prolonged 6-month PFS (41% versus 21%, *p* = 0.0003) in patients with a high-grade glioma assigned 5-ALA compared to those assigned standard operative care [89]. This study showed the usefulness of fluorescence-guided surgery for the purpose of GTR of contrast enhancement. Still, the choice of primary (GTR rate) and secondary (6-months PFS) endpoint did not permit a definitive conclusion regarding the influence of 5-ALA-guided resection on OS. Also, the standard surgical care did not include the use of neuronavigation, which is reflected by the low GTR rate in that arm. SF is a dye that accumulates in malignant glioma due to their disruption of the BBB. It is administrated by intravenous injection during surgery and, with use of a special filter in the operating microscope, results in a yellow appearance of the tumor compared to a pink appearance of the normal brain tissue. The best evidence comes from a prospective multicenter phase II trial in which 46 patients underwent SF-guided resection of a high-grade glioma that led to a GTR of contrast enhancement in 38 patients (82%). Biopsies were collected from areas with and without fluorescence in 13 of the patients and assessed for tumor presence, resulting in an 80% sensitivity and specificity [1]. The higher GTR rate in this study compared to the 5-ALA trial should be interpreted with caution since the SF study was smaller and non-randomized. Direct comparison of 5-ALA and SF is limited to small cohort studies without uniform results [16, 99]. A meta-analysis found no significant difference in the GTR rate between the agents, although it reported a much higher cost per quality added life years for 5-ALA (US$16,218) compared to SF (US$3181) [36].

Multiple studies compared 5-ALA with amino acid PET and iMRI for the detection of tumor tissue. All but one of the PET studies found a higher sensitivity for PET [20, 65] or residual post-operative tracer uptake after complete resection of 5-ALA [42, 59]. It has to be mentioned that these were all retrospective studies with different thresholds or qualitative interpretation of tracer uptake. The one study reporting 5-ALA to be more sensitive only marked deliberate residual 5-ALA fluorescence during surgery to compare with post-operative FET PET [79]. Therefore, it is unclear if there were also 5-ALA negative FET PET–positive areas. A recent meta-analysis of iMRI and 5-ALA found higher GTR rates for both techniques compared to standard operative care, yet no difference between the two techniques. They therefore concluded that a combination of 5-ALA and iMRI could have its advantages, yet future studies need to confirm this [26].

Limitation of both fluorescence agents is the lack of fluorescence in the majority of low-grade gliomas. Also, tumor tissue has been found outside the area of fluorescence of both 5-ALA and SF [28, 78, 99]. However, fluorescence-guided surgery is not limited by brain shift or navigation inaccuracy, making it therefore a suitable technique to achieve GTR of contrast enhancement in high-grade gliomas.

## Imaging for localization of brain functions and white matter tracts

### Pre-operative imaging

#### Functional MRI

**Fig. 4:**
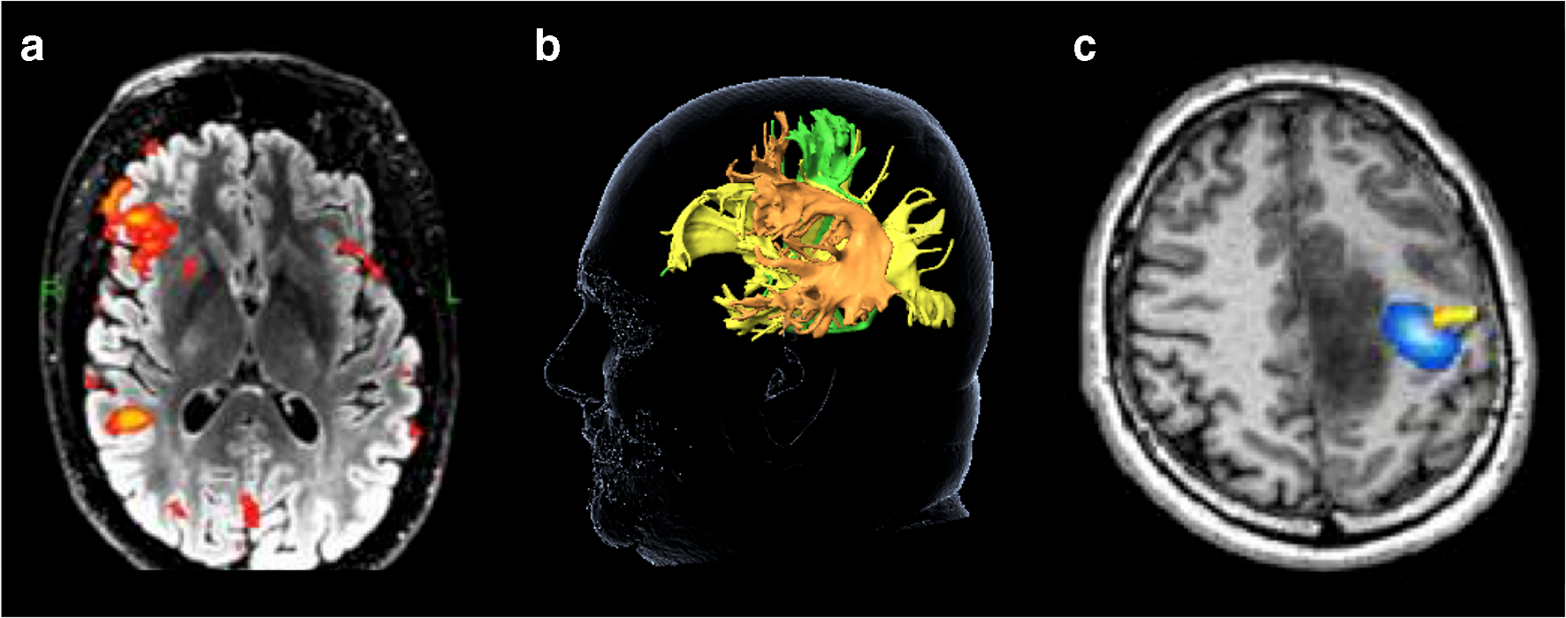
Examples of imaging for localization of brain functions and white matter tracts. **A** Functional MRI of a patient with a language located in the right hemisphere, which was confirmed with a WADA test. **B** Visualization of the left corticospinal tract (green), fasciculate arcuatus (orange) and inferior fronto-occipital fasciculus (yellow) using DTI in a patient with a left temporal diffuse astrocytoma, IDH-mutant. **C** Localization of the left motor cortex using MEG in a patient with a diffuse astrocytoma, IDH-mutant

BOLD-Functional MRI (fMRI) measures the blood oxygenation level changes caused by perfusion, which is a surrogate for neuronal activity. This allows for visualization of specific tasks-related activity such as motor function or language (Fig. 4A) [50]. Applying fMRI to localize language areas resulted in a 59–100% sensitivity and 0–97% specificity in a systematic review, with the wide ranges attributed to the heterogeneity in language tasks and imaging protocols. The authors conclude that fMRI is not an alternative for DCS language mapping [25]. Accuracy for motor function localization, compared to DCS, varies between studies, with smaller studies reporting higher accuracies (up to 100%) than the larger studies (66–72%) [3, 34, 45, 82]. In the largest study, 210 cortical sites were tested in 29 patients, resulting in an 83% sensitivity and 82% specificity. In patients with glioblastoma, however, sensitivity was only 65%, which is, according to the authors, a possible effect of the neurovascular uncoupling [7]. The limitations of fMRI have been recently described extensively and include statistical power, flexibility in data-analysis, multiple comparisons, software errors, insufficient study reporting, and lack of independent replications [56, 73]. Taken together, fMRI is not accurate enough to aid in the surgical planning, let alone replace DCS.

#### Diffusion tensor imaging

Diffusion tensor imaging (DTI) is a technique that relies on Brownian movement of water molecules in tissue. The direction of these movements is restrained in white matter fibers, which can be used to visualize the anatomical location of white matter tracts, so-called DTI tractography (Fig. 4B) [64]. This technique solemnly visualizes anatomy and does localize brain functionality. Studies comparing DTI tractography with the gold standard DCS reported mean distances between DTI tractography and positive stimulations of 5.2 to 8.7 mm [5, 27, 102]. Therefore, a minimum safe distance of 10 mm from a tract has been recommended. In an RCT comparing resection with and without pre-operative DTI in 214 patients with diffuse glioma involving the pyramidal tract, the use of DTI in patients with enhancing glioma resulted in a higher complete resection rate (74.4% versus 33.3%, *p* < 0.001), 6-month good clinical condition (70.0% versus 36.8%, *p* = 0.001), and improved median OS (21.2 versus 14.0 months, *p* = 0.048). In non-enhancing glioma patients, complete resection rate did not significantly differ, yet 6-month good clinical condition was higher in the DTI group (93.4% versus 79.1%, *p* = 0.013) [98]. Although this study clearly shows the benefit of DTI tractography in glioma resection, one must take into account that 63% of patients in the control group had poor clinical condition 6 months after surgery, which is remarkably high and would exclude these patients from adjuvant therapy in most neuro-oncology centers. High angular resolution diffusion-weighted imaging (HARDI) with q-ball algorithms is a new tractography technique with improved fiber tracking resolution at voxels with crossing fiber populations [6, 92]. One study reported that an intact left arcuate fasciculus and temporal part of the superior longitudinal fasciculus on post-operative HARDI tractography was associated with intact language, whereas an alteration or damaging of these structures resulted in, respectively, temporary or long-term language deficits [9]. Limitations of DTI are the variability of tracking algorithm settings and region-of-interest (ROI) placement. Tracking algorithm settings can lead to under- but mostly overestimation of white matter tracts. Still, 90% of the ground truth fibers are present in most of the algorithms [54]. ROI placement is subject to moderate to substantial interobserver variability but can be improved with protocols for ROI positioning [96]. Also, for HARDI DTI, considerable technical expertise is required, making it not yet available for standard practice. The high sensitivity and proven clinical value make DTI an indispensable technique for glioma surgery.

#### Magnetoencephalography

Magnetoencephalography (MEG) detects magnetic fields as result of the electric currents from neuronal activity [90]. Although this is not an imaging technique, registration of the MEG with a 3D MRI sequence allows for visualization (Fig. 4C). Like fMRI, MEG can be used for the assessment of task-based activity in the pre-operative phase. Two small studies found MEG predicted motor function areas at 4 to 17 mm from DCS sites [41, 60]. A direct comparison of MEG and fMRI for the localization of the motor cortex showed an overlap with DCS in, respectively, 100% and 73% of the patients [43], demonstrating the higher accuracy of MEG. Indirect evidence shows that if the MEG predicted functional areas within or at the margin the tumor, the EOR waspartial in 88% and complete in 12% compared to an equal division of partial and complete resections in patients with all MEG predicted functional areasoutside the tumor. Complete resection led to neurological deterioration in 2 of the 11 patients without and 2 of the 2 patients with MEG-predicted functional areas within or at the margin of the tumor, respectively [84]. On the other hand, using sensorimotor, visual, and speech MEG as a risk assessment for operation feasibility resulted in 46% of patients not to be considered for surgery due to glioma invasion of functional cortex, with only 6% of the patients who were operated suffering from neurological deterioration [22]. A major limitation of MEG is the availability, mostly in dedicated academic centers, and technical expertise needed to interpret the results. Therefore, although accuracy is considerable and the clinical application proven, MEG is not likely to become a standard modality in glioma treatment.

#### Transcranial magnetic stimulation

Transcranial magnetic stimulation (TMS), like MEG, is not an imaging technique. Due to the integration with neuronavigation (nTMS), however, it can be used to locate and visualize brain functions in the pre-operative phase. By stimulating or inhibiting cortical activity with directed magnetic fields, specific functional tasks can be localized [12]. The accuracy of nTMS, compared with DCS, for localization of the motor cortex is between 3.4–6.2 mm [44, 70]. Another function of nTMS is the combination with DTI tractography, where nTMS regions, instead of user-selected regions of interest (ROI), are used to guide the tractography. In a study comparing ROI-based with nTMS-based tractography for language pathways, respectively, 40% and 76% of the tracts were detected with DSC [61]. Using nTMS for pre-operative planning, multiple studies found a minimum distance (range 8–12 mm) from nTMS motor function or nTMS-fiber tracking that prevented any neurological deterioration [61, 81, 87]. Clinical implementation of nTMS in a large (*n* = 250) cohort of patients with brain tumors in motor eloquent locations, in comparison with a historical case-matched non-nTMS cohort, resulted in a significantly increased complete resection rate (59% versus 42%, *p* = 0.05; respectively) and increased PFS for patients with non-enhancing glioma (15.4 months versus 22.4 months, p = 0.05; respectively). Planned biopsies or non-surgical strategies were changed into resections in 68.5%, and overall post-operative deficit rate did not significantly differ between the groups (6.1% versus 8.5%; respectively) [21]. One must realize that DCS was still used in 66% of the patients and that the authors conclude that nTMS is crucial for pre-operative planning.

Only one small (*n* = 4) study used nTMS and nTMS tractography instead of DCS in patients not suitable for awake surgery with left-sided perisylvian lesions. This resulted in a GTR in all patients without any new neurological deficit with only one patient needing a second nTMS-based resection within days to achieve the GTR [32]. Overall, nTMS is a promising new technique that, combined with DTI, can overcome the ROI selection limitation of DTI and has proven its usefulness for surgical planning.

### Intraoperative imaging

#### Intraoperative MRI

Both fMRI and DTI can be acquired intraoperatively using iMRI. Intraoperative fMRI (ifMRI) can successfully localize the motor cortex during awake procedures using the task-based fMRI technique [53]. More interesting is the use of ifMRI resting state that allows for the localization of the motor cortex in patients under general anesthesia [23, 77]. Comparison of this technique with DCS in 14 patients resulted in a 62% sensitivity and 94% specificity [77]. Intraoperative DTI (iDTI) tractography has a high accuracy (100% sensitivity and 72% specificity) for the localization of the corticospinal tract, as demonstrated in a study with twenty glioma patients [35]. Another study replaced DCS with iDTI tractography for the localization of white matter tracts involved in language, resulting in a GTR in 78% and PR in 22% of the patients without any post-operative neurological deterioration [13]. The limitations of iMRI have been described above. Although ifMRI is not likely to replace DCS, iDTI has the potential to increase the safety of non-awake surgery.

## Discussion

How can imaging aid glioma surgery? We know gliomas are widespread in the brain by the time of diagnosis, so a curative resection is not possible [14]. Still, there is accumulating evidence that removing more of the tumor improves PFS and OS [8, 86]. Since glioma is an infiltrative disease, macroscopic recognition of the tumor within the normal brain can be very difficult. Imaging has the possibility to visualize the tumor and thus overcoming the macroscopic problems. Ideally, an imaging modality would not miss any tumor, 100% sensitivity, and only show tumor, 100% specificity. Unfortunately, current available imaging is not that accurate [93]. Still, using the current standard MRI to guide glioma resection has a positive impact on the treatment. Intraoperative MRI, fluorescence, and ultrasound can aid in achieving a complete resection of these standard MRI abnormalities. Since we know that gliomas extend beyond the current standard imaging abnormalities [38, 66], the next logical step is to extend the resection beyond these abnormalities. Evidence is starting to accumulate that other modalities like PET and MRSI, or FLAIR MRI in case of enhancing tumors, could guide these extended resections.

A different approach is the use of functional boundaries instead of imaging to guide a resection, whereas intraoperative mapping during awake surgery defines the limits of resection. This strategy postpones malignant transformation in LGG [17, 100]. The pitfall of functional boundaries is the choice of functions to test; removing more of the brain will lead to more deficits depending on how thoroughly the functions are tested. Translating this strategy into accuracy gives a high sensitivity and little residual tumor, but low specificity; not all resected tissue is tumor. A combination of image-guided extended resection and intraoperative stimulation mapping could improve the specificity by removing less normal brain, while keeping a high sensitivity.

## Future directions

Randomized clinical trials are needed to compare the influence of image-guided glioma resection, possibly with addition of intraoperative fluorescence, based on standard MRI versus the most optimal imaging. In order to determine the most optimal imaging for the detection of glioma infiltration, studies directly comparing different imaging modalities, MRI sequences, and combinations of imaging, such as the FRONTIER study [94], have to be conducted. Besides pre-operative imaging, intraoperative ultrasound has demonstrated great potential and the results of the randomized US-GLIOMA trial (NCT03531333) are awaited for. Extending the resection implies that more frequently, functional areas will be encountered. DCS remains the gold standard for the localization of important brain functions and white matter tracts. Imaging, however, is indispensable for surgical planning, including the choice of awake versus non-awake surgery. DTI has proven its clinical value in an RCT, and studies exploring the increased accuracy of HARDI DTI, possibly in combination with nTMS, are needed.

## Data Availability

Not applicable.

## References

[1] Acerbi F, Broggi M, Schebesch KM, Hohne J, Cavallo C, De Laurentis C, Eoli M, Anghileri E, Servida M, Boffano C, Pollo B, Schiariti M, Visintini S, Montomoli C, Bosio L, La Corte E, Broggi G, Brawanski A, Ferroli P (2018). Fluorescein-guided surgery for resection of high-grade gliomas: a multicentric prospective phase II study (FLUOGLIO). Clin Cancer Res.

[2] Aghi MK, Nahed BV, Sloan AE, Ryken TC, Kalkanis SN, Olson JJ (2015). The role of surgery in the management of patients with diffuse low grade glioma: a systematic review and evidence-based clinical practice guideline. J Neuro-Oncol.

[3] Bartos R, Jech R, Vymazal J, Petrovicky P, Vachata P, Hejcl A, Zolal A, Sames M (2009). Validity of primary motor area localization with fMRI versus electric cortical stimulation: a comparative study. Acta Neurochir.

[4] Ben Abdallah M, Blonski M, Wantz-Mezieres S, Gaudeau Y, Taillandier L, Moureaux JM (2016). Statistical evaluation of manual segmentation of a diffuse low-grade glioma MRI dataset. Conference proceedings : Annual International Conference of the IEEE Engineering in Medicine and Biology Society IEEE Engineering in Medicine and Biology Society Annual Conference.

[5] Berman JI, Berger MS, Chung SW, Nagarajan SS, Henry RG (2007). Accuracy of diffusion tensor magnetic resonance imaging tractography assessed using intraoperative subcortical stimulation mapping and magnetic source imaging. J Neurosurg.

[6] Berman JI, Chung S, Mukherjee P, Hess CP, Han ET, Henry RG (2008). Probabilistic streamline q-ball tractography using the residual bootstrap. NeuroImage.

[7] Bizzi A, Blasi V, Falini A, Ferroli P, Cadioli M, Danesi U, Aquino D, Marras C, Caldiroli D, Broggi G (2008). Presurgical functional MR imaging of language and motor functions: validation with intraoperative electrocortical mapping. Radiology.

[8] Brown TJ, Brennan MC, Li M, Church EW, Brandmeir NJ, Rakszawski KL, Patel AS, Rizk EB, Suki D, Sawaya R, Glantz M (2016). Association of the extent of resection with survival in glioblastoma: a systematic review and meta-analysis. JAMA oncology.

[9] Caverzasi E, Hervey-Jumper SL, Jordan KM, Lobach IV, Li J, Panara V, Racine CA, Sankaranarayanan V, Amirbekian B, Papinutto N, Berger MS, Henry RG (2016). Identifying preoperative language tracts and predicting postoperative functional recovery using HARDI q-ball fiber tractography in patients with gliomas. J Neurosurg.

[10] Coburger J, Konig RW, Scheuerle A, Engelke J, Hlavac M, Thal DR, Wirtz CR (2014). Navigated high frequency ultrasound: description of technique and clinical comparison with conventional intracranial ultrasound. World neurosurgery.

[11] Coburger J, Scheuerle A, Kapapa T, Engelke J, Thal DR, Wirtz CR, Konig R (2015). Sensitivity and specificity of linear array intraoperative ultrasound in glioblastoma surgery: a comparative study with high field intraoperative MRI and conventional sector array ultrasound. Neurosurg Rev.

[12] Curra A, Modugno N, Inghilleri M, Manfredi M, Hallett M, Berardelli A (2002). Transcranial magnetic stimulation techniques in clinical investigation. Neurology.

[13] D'Andrea G, Familiari P, Di Lauro A, Angelini A, Sessa G (2016). Safe resection of gliomas of the dominant angular gyrus availing of preoperative FMRI and intraoperative DTI: preliminary series and surgical technique. World neurosurgery.

[14] Dandy WE (1928). Removal of right cerebral hemisphere for certain tumors with hemiplegia. JAMA.

[15] De Witt Hamer PC, Robles SG, Zwinderman AH, Duffau H, Berger MS (2012). Impact of intraoperative stimulation brain mapping on glioma surgery outcome: a meta-analysis. Journal of clinical oncology : official journal of the American Society of Clinical Oncology.

[16] Della Puppa A, Munari M, Gardiman MP, Volpin F (2019). Combined fluorescence using 5- aminolevulinic acid and fluorescein sodium at glioblastoma border: intraoperative findings and histopathologic data about 3 newly diagnosed consecutive cases. World neurosurgery.

[17] Duffau H (2016). Long-term outcomes after supratotal resection of diffuse low-grade gliomas: a consecutive series with 11-year follow-up. Acta Neurochir.

[18] Ellingson BM, Bendszus M, Boxerman J, Barboriak D, Erickson BJ, Smits M, Nelson SJ, Gerstner E, Alexander B, Goldmacher G, Wick W, Vogelbaum M, Weller M, Galanis E, Kalpathy-Cramer J, Shankar L, Jacobs P, Pope WB, Yang D, Chung C, Knopp MV, Cha S, van den Bent MJ, Chang S, Yung WK, Cloughesy TF, Wen PY, Gilbert MR, Jumpstarting Brain Tumor Drug Development Coalition Imaging Standardization Steering C (2015) Consensus recommendations for a standardized brain tumor imaging protocol in clinical trials. Neuro-oncology 17:1188–1198. 10.1093/neuonc/nov095 10.1093/neuonc/nov095 PMC4588759 26250565

[19] Ening G, Osterheld F, Capper D, Schmieder K, Brenke C (2015). Risk factors for glioblastoma therapy associated complications. Clin Neurol Neurosurg.

[20] Floeth FW, Sabel M, Ewelt C, Stummer W, Felsberg J, Reifenberger G, Steiger HJ, Stoffels G, Coenen HH, Langen KJ (2011). Comparison of (18)F-FET PET and 5-ALA fluorescence in cerebral gliomas. Eur J Nucl Med Mol Imaging.

[21] Frey D, Schilt S, Strack V, Zdunczyk A, Rosler J, Niraula B, Vajkoczy P, Picht T (2014). Navigated transcranial magnetic stimulation improves the treatment outcome in patients with brain tumors in motor eloquent locations. Neuro-oncology.

[22] Ganslandt O, Buchfelder M, Hastreiter P, Grummich P, Fahlbusch R, Nimsky C (2004). Magnetic source imaging supports clinical decision making in glioma patients. Clin Neurol Neurosurg.

[23] Gasser T, Ganslandt O, Sandalcioglu E, Stolke D, Fahlbusch R, Nimsky C (2005). Intraoperative functional MRI: implementation and preliminary experience. NeuroImage.

[24] Gill BJ, Pisapia DJ, Malone HR, Goldstein H, Lei L, Sonabend A, Yun J, Samanamud J, Sims JS, Banu M, Dovas A, Teich AF, Sheth SA, McKhann GM, Sisti MB, Bruce JN, Sims PA, Canoll P (2014). MRI-localized biopsies reveal subtype-specific differences in molecular and cellular composition at the margins of glioblastoma. Proc Natl Acad Sci U S A.

[25] Giussani C, Roux FE, Ojemann J, Sganzerla EP, Pirillo D, Papagno C (2010). Is preoperative functional magnetic resonance imaging reliable for language areas mapping in brain tumor surgery? Review of language functional magnetic resonance imaging and direct cortical stimulation correlation studies. Neurosurgery.

[26] Golub D, Hyde J, Dogra S, Nicholson J, Kirkwood KA, Gohel P, Loftus S, Schwartz TH (2020) Intraoperative MRI versus 5-ALA in high-grade glioma resection: a network meta-analysis. J Neurosurg:1–15. 10.3171/2019.12.JNS191203 10.3171/2019.12.JNS191203 32084631

[27] Guo J, Yao C, Chen H, Zhuang D, Tang W, Ren G, Wang Y, Wu J, Huang F, Zhou L (2012). The relationship between Cho/NAA and glioma metabolism: implementation for margin delineation of cerebral gliomas. Acta Neurochir.

[28] Hauser SB, Kockro RA, Actor B, Sarnthein J, Bernays RL (2016). Combining 5-aminolevulinic acid fluorescence and intraoperative magnetic resonance imaging in glioblastoma surgery: a histology-based evaluation. Neurosurgery.

[29] Heinzel A, Stock S, Langen KJ, Muller D (2012). Cost-effectiveness analysis of amino acid PET-guided surgery for supratentorial high-grade gliomas. Journal of nuclear medicine : official publication, Society of Nuclear Medicine.

[30] Henker C, Hiepel MC, Kriesen T, Scherer M, Glass A, Herold-Mende C, Bendszus M, Langner S, Weber MA, Schneider B, Unterberg A, Piek J (2019). Volumetric assessment of glioblastoma and its predictive value for survival. Acta Neurochir.

[31] Hutton BF, Braun M (2003). Software for image registration: algorithms, accuracy, efficacy. Semin Nucl Med.

[32] Ille S, Sollmann N, Butenschoen VM, Meyer B, Ringel F, Krieg SM (2016). Resection of highly language-eloquent brain lesions based purely on rTMS language mapping without awake surgery. Acta Neurochir.

[33] Ius T, Isola M, Budai R, Pauletto G, Tomasino B, Fadiga L, Skrap M (2012). Low-grade glioma surgery in eloquent areas: volumetric analysis of extent of resection and its impact on overall survival. A single-institution experience in 190 patients: clinical article. J Neurosurg.

[34] Jack CR, Thompson RM, Butts RK, Sharbrough FW, Kelly PJ, Hanson DP, Riederer SJ, Ehman RL, Hangiandreou NJ, Cascino GD (1994). Sensory motor cortex: correlation of presurgical mapping with functional MR imaging and invasive cortical mapping. Radiology.

[35] Javadi SA, Nabavi A, Giordano M, Faghihzadeh E, Samii A (2017). Evaluation of diffusion tensor Imaging-based tractography of the corticospinal tract: a correlative study with intraoperative magnetic resonance imaging and direct electrical subcortical stimulation. Neurosurgery.

[36] Kaneko S, Eljamel MS (2017). Fluorescence image-guided neurosurgery. Future Oncol.

[37] Keles GE, Chang EF, Lamborn KR, Tihan T, Chang CJ, Chang SM, Berger MS (2006). Volumetric extent of resection and residual contrast enhancement on initial surgery as predictors of outcome in adult patients with hemispheric anaplastic astrocytoma. J Neurosurg.

[38] Kelly PJ, Daumas-Duport C, Kispert DB, Kall BA, Scheithauer BW, Illig JJ (1987). Imaging-based stereotaxic serial biopsies in untreated intracranial glial neoplasms. J Neurosurg.

[39] Kelly PJ, Daumas-Duport C, Scheithauer BW, Kall BA, Kispert DB (1987). Stereotactic histologic correlations of computed tomography- and magnetic resonance imaging-defined abnormalities in patients with glial neoplasms. Mayo Clin Proc.

[40] Keppler JS, Conti PS (2001). A cost analysis of positron emission tomography. AJR Am J Roentgenol.

[41] Kirsch HE, Zhu Z, Honma S, Findlay A, Berger MS, Nagarajan SS (2007). Predicting the location of mouth motor cortex in patients with brain tumors by using somatosensory evoked field measurements. J Neurosurg.

[42] Klasner B, Buchmann N, Gempt J, Ringel F, Lapa C, Krause BJ (2015). Early [18F]FET-PET in gliomas after surgical resection: comparison with MRI and histopathology. PLoS One.

[43] Korvenoja A, Kirveskari E, Aronen HJ, Avikainen S, Brander A, Huttunen J, Ilmoniemi RJ, Jaaskelainen JE, Kovala T, Makela JP, Salli E, Seppa M (2006). Sensorimotor cortex localization: comparison of magnetoencephalography, functional MR imaging, and intraoperative cortical mapping. Radiology.

[44] Krieg SM, Shiban E, Buchmann N, Meyer B, Ringel F (2013). Presurgical navigated transcranial magnetic brain stimulation for recurrent gliomas in motor eloquent areas. Clinical neurophysiology : official journal of the International Federation of Clinical Neurophysiology.

[45] Krings T, Schreckenberger M, Rohde V, Spetzger U, Sabri O, Reinges MH, Hans FJ, Meyer PT, Moller-Hartmann W, Gilsbach JM, Buell U, Thron A (2002). Functional MRI and 18F FDG-positron emission tomography for presurgical planning: comparison with electrical cortical stimulation. Acta Neurochir.

[46] Kubben P (2014) Ultra low-field strength intraoperative mri for glioblastoma surgery. Universiteit Maastricht

[47] Kubben PL, Postma AA, Kessels AG, van Overbeeke JJ, van Santbrink H (2010). Intraobserver and interobserver agreement in volumetric assessment of glioblastoma multiforme resection. Neurosurgery.

[48] Kubben PL, Scholtes F, Schijns OE, Ter Laak-Poort MP, Teernstra OP, Kessels AG, van Overbeeke JJ, Martin DH, van Santbrink H (2014). Intraoperative magnetic resonance imaging versus standard neuronavigation for the neurosurgical treatment of glioblastoma: a randomized controlled trial. Surg Neurol Int.

[49] Langen KJ, Galldiks N, Hattingen E, Shah NJ (2017). Advances in neuro-oncology imaging. Nat Rev Neurol.

[50] Le Bihan D, Jezzard P, Haxby J, Sadato N, Rueckert L, Mattay V (1995). Functional magnetic resonance imaging of the brain. Ann Intern Med.

[51] Li Y, Osorio JA, Ozturk-Isik E, Chen AP, Xu D, Crane JC, Cha S, Chang S, Berger MS, Vigneron DB, Nelson SJ (2006). Considerations in applying 3D PRESS H-1 brain MRSI with an eight-channel phased-array coil at 3 T. Magn Reson Imaging.

[52] Li YM, Suki D, Hess K, Sawaya R (2016). The influence of maximum safe resection of glioblastoma on survival in 1229 patients: can we do better than gross-total resection?. J Neurosurg.

[53] Lu J, Wu J, Yao C, Zhuang D, Qiu T, Hu X, Zhang J, Gong X, Liang W, Mao Y, Zhou L (2013). Awake language mapping and 3-Tesla intraoperative MRI-guided volumetric resection for gliomas in language areas. Journal of clinical neuroscience : official journal of the Neurosurgical Society of Australasia.

[54] Maier-Hein KH, Neher PF, Houde JC, Cote MA, Garyfallidis E, Zhong J, Chamberland M, Yeh FC, Lin YC, Ji Q, Reddick WE, Glass JO, Chen DQ, Feng Y, Gao C, Wu Y, Ma J, Renjie H, Li Q, Westin CF, Deslauriers-Gauthier S, Gonzalez JOO, Paquette M, St-Jean S, Girard G, Rheault F, Sidhu J, Tax CMW, Guo F, Mesri HY, David S, Froeling M, Heemskerk AM, Leemans A, Bore A, Pinsard B, Bedetti C, Desrosiers M, Brambati S, Doyon J, Sarica A, Vasta R, Cerasa A, Quattrone A, Yeatman J, Khan AR, Hodges W, Alexander S, Romascano D, Barakovic M, Auria A, Esteban O, Lemkaddem A, Thiran JP, Cetingul HE, Odry BL, Mailhe B, Nadar MS, Pizzagalli F, Prasad G, Villalon-Reina JE, Galvis J, Thompson PM, Requejo FS, Laguna PL, Lacerda LM, Barrett R, Dell'Acqua F, Catani M, Petit L, Caruyer E, Daducci A, Dyrby TB, Holland-Letz T, Hilgetag CC, Stieltjes B, Descoteaux M (2017). The challenge of mapping the human connectome based on diffusion tractography. Nat Commun.

[55] Makary M, Chiocca EA, Erminy N, Antor M, Bergese SD, Abdel-Rasoul M, Fernandez S, Dzwonczyk R (2011). Clinical and economic outcomes of low-field intraoperative MRI-guided tumor resection neurosurgery. Journal of magnetic resonance imaging : JMRI.

[56] Mandonnet E, Duffau H (2017) Chapter 5. In: Moliterno Gunel J, Piepmeier JM, Baehring JM (eds) Malignant Brain Tumors: State-of-the-Art Treatment. doi:10.1007/978-3-319-49864-5

[57] Martin C, Alexander E, Wong T, Schwartz R, Jolesz F, Black PM (1998). Surgical treatment of low-grade gliomas in the intraoperative magnetic resonance imager. Neurosurg Focus.

[58] Mohammadi AM, Sullivan TB, Barnett GH, Recinos V, Angelov L, Kamian K, Vogelbaum MA (2014) Use of high-field intraoperative magnetic resonance imaging to enhance the extent of resection of enhancing and nonenhancing gliomas. Neurosurgery 74:339–348; **discussion 349** . 10.1227/NEU.0000000000000278**quiz 349-350** 10.1227/NEU.0000000000000278 24368543

[59] Muther M, Koch R, Weckesser M, Sporns P, Schwindt W, Stummer W (2019). 5-Aminolevulinic acid fluorescence-guided resection of 18F-FET-PET positive tumor beyond gadolinium enhancing tumor improves survival in glioblastoma. Neurosurgery.

[60] Nagarajan S, Kirsch H, Lin P, Findlay A, Honma S, Berger MS (2008). Preoperative localization of hand motor cortex by adaptive spatial filtering of magnetoencephalography data. J Neurosurg.

[61] Negwer C, Sollmann N, Ille S, Hauck T, Maurer S, Kirschke JS, Ringel F, Meyer B, Krieg SM (2017). Language pathway tracking: comparing nTMS-based DTI fiber tracking with a cubic ROIs-based protocol. J Neurosurg.

[62] Nimsky C, Ganslandt O, Hastreiter P, Wang R, Benner T, Sorensen AG, Fahlbusch R (2005). Preoperative and intraoperative diffusion tensor imaging-based fiber tracking in glioma surgery. Neurosurgery.

[63] Nimsky C, Ganslandt O, Von Keller B, Romstock J, Fahlbusch R (2004). Intraoperative high-field-strength MR imaging: implementation and experience in 200 patients. Radiology.

[64] Nucifora PG, Verma R, Lee SK, Melhem ER (2007). Diffusion-tensor MR imaging and tractography: exploring brain microstructure and connectivity. Radiology.

[65] Pala A, Reske SN, Eberhardt N, Scheuerle A, Konig R, Schmitz B, Beer AJ, Wirtz CR, Coburger J (2019). Diagnostic accuracy of intraoperative perfusion-weighted MRI and 5-aminolevulinic acid in relation to contrast-enhanced intraoperative MRI and (11)C-methionine positron emission tomography in resection of glioblastoma: a prospective study. Neurosurg Rev.

[66] Pallud J, Varlet P, Devaux B, Geha S, Badoual M, Deroulers C, Page P, Dezamis E, Daumas-Duport C, Roux FX (2010). Diffuse low-grade oligodendrogliomas extend beyond MRI-defined abnormalities. Neurology.

[67] Pamir MN, Ozduman K, Dincer A, Yildiz E, Peker S, Ozek MM (2010). First intraoperative, shared-resource, ultrahigh-field 3-tesla magnetic resonance imaging system and its application in low-grade glioma resection. J Neurosurg.

[68] Pessina F, Navarria P, Cozzi L, Ascolese AM, Simonelli M, Santoro A, Clerici E, Rossi M, Scorsetti M, Bello L (2017). Maximize surgical resection beyond contrast-enhancing boundaries in newly diagnosed glioblastoma multiforme: is it useful and safe? A single institution retrospective experience. J Neuro-Oncol.

[69] Pichlmeier U, Bink A, Schackert G, Stummer W, Group ALAGS (2008). Resection and survival in glioblastoma multiforme: an RTOG recursive partitioning analysis of ALA study patients. Neuro-oncology.

[70] Picht T, Mularski S, Kuehn B, Vajkoczy P, Kombos T, Suess O (2009). Navigated transcranial magnetic stimulation for preoperative functional diagnostics in brain tumor surgery. Neurosurgery.

[71] Pirotte B, Goldman S, Dewitte O, Massager N, Wikler D, Lefranc F, Ben Taib NO, Rorive S, David P, Brotchi J, Levivier M (2006). Integrated positron emission tomography and magnetic resonance imaging-guided resection of brain tumors: a report of 103 consecutive procedures. J Neurosurg.

[72] Pirotte BJ, Levivier M, Goldman S, Massager N, Wikler D, Dewitte O, Bruneau M, Rorive S, David P, Brotchi J (2009). Positron emission tomography-guided volumetric resection of supratentorial high-grade gliomas: a survival analysis in 66 consecutive patients. Neurosurgery.

[73] Poldrack RA, Baker CI, Durnez J, Gorgolewski KJ, Matthews PM, Munafo MR, Nichols TE, Poline JB, Vul E, Yarkoni T (2017). Scanning the horizon: towards transparent and reproducible neuroimaging research. Nat Rev Neurosci.

[74] Prada F, Del Bene M, Mattei L, Casali C, Filippini A, Legnani F, Mangraviti A, Saladino A, Perin A, Richetta C, Vetrano I, Moiraghi A, Saini M, DiMeco F (2014). Fusion imaging for intra-operative ultrasound-based navigation in neurosurgery. Journal of ultrasound.

[75] Prada F, Vitale V, Del Bene M, Boffano C, Sconfienza LM, Pinzi V, Mauri G, Solbiati L, Sakas G, Kolev V, D'Incerti L, DiMeco F (2017). Contrast-enhanced MR imaging versus contrast-enhanced US: a comparison in glioblastoma surgery by using intraoperative fusion Imaging. Radiology.

[76] Price SJ, Jena R, Burnet NG, Hutchinson PJ, Dean AF, Pena A, Pickard JD, Carpenter TA, Gillard JH (2006). Improved delineation of glioma margins and regions of infiltration with the use of diffusion tensor imaging: an image-guided biopsy study. AJNR Am J Neuroradiol.

[77] Qiu TM, Gong FY, Gong X, Wu JS, Lin CP, Biswal BB, Zhuang DX, Yao CJ, Zhang XL, Lu JF, Zhu FP, Mao Y, Zhou LF (2017). Real-time motor cortex mapping for the safe resection of glioma: an intraoperative resting-state fMRI study. AJNR Am J Neuroradiol.

[78] Roberts DW, Valdes PA, Harris BT, Fontaine KM, Hartov A, Fan X, Ji S, Lollis SS, Pogue BW, Leblond F, Tosteson TD, Wilson BC, Paulsen KD (2011). Coregistered fluorescence-enhanced tumor resection of malignant glioma: relationships between delta-aminolevulinic acid-induced protoporphyrin IX fluorescence, magnetic resonance imaging enhancement, and neuropathological parameters. Clinical article. Journal of neurosurgery.

[79] Roessler K, Becherer A, Donat M, Cejna M, Zachenhofer I (2012). Intraoperative tissue fluorescence using 5-aminolevolinic acid (5-ALA) is more sensitive than contrast MRI or amino acid positron emission tomography ((18)F-FET PET) in glioblastoma surgery. Neurol Res.

[80] Romano A, D'Andrea G, Calabria LF, Coppola V, Espagnet CR, Pierallini A, Ferrante L, Fantozzi L, Bozzao A (2011). Pre- and intraoperative tractographic evaluation of corticospinal tract shift. Neurosurgery.

[81] Rosenstock T, Giampiccolo D, Schneider H, Runge SJ, Bahrend I, Vajkoczy P, Picht T (2017). Specific DTI seeding and diffusivity-analysis improve the quality and prognostic value of TMS-based deterministic DTI of the pyramidal tract. NeuroImage Clinical.

[82] Roux FE, Boulanouar K, Ranjeva JP, Tremoulet M, Henry P, Manelfe C, Sabatier J, Berry I (1999). Usefulness of motor functional MRI correlated to cortical mapping in Rolandic low-grade astrocytomas. Acta Neurochir.

[83] Sastry R, Bi WL, Pieper S, Frisken S, Kapur T, Wells W, Golby AJ (2017). Applications of ultrasound in the resection of brain tumors. Journal of neuroimaging : official journal of the American Society of Neuroimaging.

[84] Schiffbauer H, Ferrari P, Rowley HA, Berger MS, Roberts TP (2001). Functional activity within brain tumors: a magnetic source imaging study. Neurosurgery.

[85] Senft C, Bink A, Franz K, Vatter H, Gasser T, Seifert V (2011). Intraoperative MRI guidance and extent of resection in glioma surgery: a randomised, controlled trial. The Lancet Oncology.

[86] Smith JS, Chang EF, Lamborn KR, Chang SM, Prados MD, Cha S, Tihan T, Vandenberg S, McDermott MW, Berger MS (2008). Role of extent of resection in the long-term outcome of low-grade hemispheric gliomas. Journal of clinical oncology : official journal of the American Society of Clinical Oncology.

[87] Sollmann N, Wildschuetz N, Kelm A, Conway N, Moser T, Bulubas L, Kirschke JS, Meyer B, Krieg SM (2018). Associations between clinical outcome and navigated transcranial magnetic stimulation characteristics in patients with motor-eloquent brain lesions: a combined navigated transcranial magnetic stimulation-diffusion tensor imaging fiber tracking approach. J Neurosurg.

[88] Steinmeier R, Rachinger J, Kaus M, Ganslandt O, Huk W, Fahlbusch R (2000). Factors influencing the application accuracy of neuronavigation systems. Stereotact Funct Neurosurg.

[89] Stummer W, Pichlmeier U, Meinel T, Wiestler OD, Zanella F, Reulen HJ, Group AL-GS (2006). Fluorescence-guided surgery with 5-aminolevulinic acid for resection of malignant glioma: a randomised controlled multicentre phase III trial. The Lancet Oncology.

[90] Sutherling WW, Crandall PH, Darcey TM, Becker DP, Levesque MF, Barth DS (1988). The magnetic and electric fields agree with intracranial localizations of somatosensory cortex. Neurology.

[91] Tanaka S, Puffer RC, Hoover JM, Goerss SJ, Haugen LM, McGee K, Parney IF (2012) Increased frameless stereotactic accuracy with high-field intraoperative magnetic resonance imaging Neurosurgery 71:ons321-327; discussion ons327-328. doi:10.1227/NEU.0b013e31826a88a9 10.1227/NEU.0b013e31826a88a9 22843131

[92] Tuch DS (2004). Q-ball imaging. Magn Reson Med.

[93] Verburg N, Hoefnagels FWA, Barkhof F, Boellaard R, Goldman S, Guo J, Heimans JJ, Hoekstra OS, Jain R, Kinoshita M, Pouwels PJW, Price SJ, Reijneveld JC, Stadlbauer A, Vandertop WP, Wesseling P, Zwinderman AH, De Witt Hamer PC (2017). Diagnostic accuracy of neuroimaging to delineate diffuse gliomas within the brain: a meta-analysis. AJNR Am J Neuroradiol.

[94] Verburg N, Koopman T, Yaqub MM, Hoekstra OS, Lammertsma AA, Barkhof F, Pouwels PJW, Reijneveld JC, Heimans JJ, Rozemuller AJM, Bruynzeel AME, Lagerwaard F, Vandertop WP, Boellaard R, Wesseling P, de Witt Hamer PC (2020). Improved detection of diffuse glioma infiltration with imaging combinations: a diagnostic accuracy study. Neuro-oncology.

[95] Visser M, Muller DMJ, van Duijn RJM, Smits M, Verburg N, Hendriks EJ, Nabuurs RJA, Bot JCJ, Eijgelaar RS, Witte M, van Herk MB, Barkhof F, de Witt Hamer PC, de Munck JC (2019). Inter-rater agreement in glioma segmentations on longitudinal MRI. NeuroImage Clinical.

[96] Wakana S, Caprihan A, Panzenboeck MM, Fallon JH, Perry M, Gollub RL, Hua K, Zhang J, Jiang H, Dubey P, Blitz A, van Zijl P, Mori S (2007). Reproducibility of quantitative tractography methods applied to cerebral white matter. NeuroImage.

[97] Wang J, Liu X, Ba YM, Yang YL, Gao GD, Wang L, Duan YY (2012). Effect of sonographically guided cerebral glioma surgery on survival time. Journal of ultrasound in medicine : official journal of the American Institute of Ultrasound in Medicine.

[98] Wu JS, Zhou LF, Tang WJ, Mao Y, Hu J, Song YY, Hong XN, Du GH (2007). Clinical evaluation and follow-up outcome of diffusion tensor imaging-based functional neuronavigation: a prospective, controlled study in patients with gliomas involving pyramidal tracts. Neurosurgery.

[99] Yano H, Nakayama N, Ohe N, Miwa K, Shinoda J, Iwama T (2017). Pathological analysis of the surgical margins of resected glioblastomas excised using photodynamic visualization with both 5-aminolevulinic acid and fluorescein sodium. J Neuro-Oncol.

[100] Yordanova YN, Moritz-Gasser S, Duffau H (2011). Awake surgery for WHO grade II gliomas within “noneloquent” areas in the left dominant hemisphere: toward a “supratotal” resection. Clinical article Journal of neurosurgery.

[101] Zhang J, Zhuang DX, Yao CJ, Lin CP, Wang TL, Qin ZY, Wu JS (2016). Metabolic approach for tumor delineation in glioma surgery: 3D MR spectroscopy image-guided resection. J Neurosurg.

[102] Zolal A, Hejcl A, Vachata P, Bartos R, Humhej I, Malucelli A, Novakova M, Hrach K, Derner M, Sames M (2012). The use of diffusion tensor images of the corticospinal tract in intrinsic brain tumor surgery: a comparison with direct subcortical stimulation. Neurosurgery.

